# Open Appendicectomy under Spinal Anesthesia—A Valuable Alternative during COVID-19

**DOI:** 10.1055/s-0041-1725933

**Published:** 2021-06-03

**Authors:** Dinh Van Chi Mai, Alex Sagar, Oliver Claydon, Ji Young Park, Niteen Tapuria, Barrie D. Keeler

**Affiliations:** 1 Department of Colorectal Surgery, Milton Keynes University Hospital, Standing Way, Milton Keynes, United Kingdom; 2 Department of General Surgery, Milton Keynes University Hospital, Standing Way, Milton Keynes, United Kingdom

**Keywords:** COVID-19, SARS-CoV-2, appendicitis, appendicectomy, anesthesia, spinal

## Abstract

**Introduction** Concerns relating to coronavirus disease 2019 (COVID-19) and general anesthesia (GA) prompted our department to consider that open appendicectomy under spinal anesthesia (SA) avoids aerosolization from intubation and laparoscopy. While common in developing nations, it is unusual in the United Kingdom. We present the first United Kingdom case series and discuss its potential role during and after this pandemic.

**Methods** We prospectively studied patients with appendicitis at a British district general hospital who were unsuitable for conservative management and consequently underwent open appendicectomy under SA. We also reviewed patient satisfaction after 30 days. This ran for 5 weeks from March 25th, 2020 until the surgical department reverted to the laparoscopic appendicectomy as the standard of care. Main outcomes were 30-day complication rates and patient satisfaction.

**Results** None of the included seven patients were COVID positive. The majority (four-sevenths) had complicated appendicitis. There were no major adverse (Clavien-Dindo grade III to V) postoperative events. Two patients suffered minor postoperative complications. Two experienced intraoperative pain. Mean operative time was 44 minutes. Median length of stay and return to activity was 1 and 14 days, respectively. Although four stated preference in hindsight for GA, the majority (five-sevenths) were satisfied with the operative experience under SA.

**Discussion** Although contraindications, risk of pain, and specific complications may be limiting, our series demonstrates open appendicectomy under SA to be safe and feasible in the United Kingdom. The technique could be a valuable contingency for COVID-suspected cases and patients with high-risk respiratory disease.

The United Kingdom's severe acute respiratory syndrome coronavirus 2 (SARS-COV-2) outbreak forced surgical departments in the United Kingdom to change the way they operate. An international cohort study showed the 30-day mortality rate was 23.8% for patients undergoing surgery with perioperative COVID-19 infection. 1 Conservative management was therefore advised during the outbreak but there would inevitably still be a need for surgery, and it was therefore crucial that surgeons and anesthetists had a broad range of operative techniques at their disposal to minimize adverse outcomes for patients and staff.

In the United Kingdom, laparoscopic appendicectomy has generally been adopted as the standard of care for appendicitis since 2014 as outlined by the joint commissioning guidelines published by Royal Colleges of Surgeons (RCS) and Association of Surgeons of Great Britain and Ireland. 2 On March 25th, 2020, the Intercollegiate RCS General Surgery guidance advised considering open appendicectomy or conservative management 3 due to the impact of COVID-19. SARS-COV-2 remains aerosolized for 3 hours 4 and while tracheal intubation is known to be aerosol generating, 5 the extent to which this applies to laparoscopy is less clear. However, as it has been demonstrated that other viruses become aerosolized during laparoscopic procedures 6 and SARS-COV-2 has been detected in feces and peritoneal fluid, 7 8 it is postulated that exposure of the peritoneal cavity to positive pressure and risk of fecal contamination during laparoscopic appendicectomy could pose infection control risks. This is further complicated by challenges in determining the COVID status in acute surgical patients due to asymptomatic infection and false negative rates swab testing. 9

We hypothesized that an anesthetic and operative approach avoiding tracheal intubation and laparoscopy for appendicectomy could be beneficial for patients and staff. Open appendicectomy under spinal anesthesia (SA) is one such technique but it lacks in terms of high-quality research on outcomes, with little data originating from health care systems comparable to the United Kingdom. We therefore present a series of seven patients undergoing open appendicectomy under SA during COVID-19.

## Methods

### Study Design

A prospective case series of open appendicectomy under SA during the COVID-19 pandemic was performed in a British district general hospital. This commenced on March 25th, 2020 corresponding with the release of the first Intercollegiate RCS guidance. 3 Initial departmental practice was to treat clinically uncomplicated appendicitis conservatively and reserve surgery for suspected complicated disease or failed nonoperative management. End point was the reinstatement of laparoscopic appendicectomy as the standard of care either locally or nationally. A follow-up patient survey was conducted after 30 postoperative days.

The project was approved by our Research and Development department and registered as project 498. Ethical approval was not required as changes in practice were governed by clinical decisions based on professional guidance rather than for research purposes; patient satisfaction was service evaluation of such changes.

### Participants

All open appendicectomies under SA were included from this period; planned GA or laparoscopic surgery was excluded. This decision for SA was clinical, made by consultant surgeons and anesthetists independent of this study. Relative contraindications for SA were patient choice; generalized peritonitis, signs of systemic sepsis, or radiological suggestion of perforation.

### Outcomes

Primary outcome was 30-day postoperative complications using the Clavien-Dindo (C-D) grading system. 10

Secondary outcomes were length of stay (LOS); readmissions; intraoperative pain; a Likert-type pain score 24 hours postoperatively; time to normal activity; patient satisfaction; and preference for GA or SA in hindsight.

## Results

The series ran from March 25, 2020 until April 30, 2020. After this, the department returned to laparoscopic appendicectomy as first-line treatment for appendicitis due to the locally low COVID-19 caseload, with open appendicectomy then reserved only for patients with suspected or confirmed COVID-19 infection. There were 19 episodes of appendicitis during this period. Eight patients were treated conservatively; 11 underwent open appendicectomy; four of these had GA because of relative contraindications to SA. The remaining seven underwent open appendicectomy under SA and were included in the series.

### Preoperative Characteristics

There were six males and one female with a median age of 35 years (range, 24–56). Only one had major co-morbidities (obstructive sleep apnea). Table 1 shows the mean biochemical markers and physiological parameters.

**Table 1:** Patient characteristics

Characteristics (i)	
Duration of symptoms (hours)	40
Heart rate (beats-per-minute)	90
Respiratory rate (breaths-per-minute)	17
Systolic blood pressure (mm Hg)	129
Oxygen saturations on room air (%)	97
Temperature (degrees Celsius)	37.3
White blood cell count (10 ^9^ /L)	13.1
C-reactive protein (mg/L)	89.0

Note: Data expressed as the mean.

Six of seven underwent preoperative computed tomography scanning, providing radiological confirmation of appendicitis.

No patients were suspected to have COVID-19 infection. Six patients received preoperative chest imaging which showed no COVID-19 like changes.

### Operative Characteristics

All patients underwent SA with bupivacaine. Six of seven patients also received intrathecal diamorphine or fentanyl. The one patient who did not receive these later required intraoperative intravenous (IV) fentanyl.

Mean anesthetic preparation and operating times were 23 and 44 minutes, respectively. Patients received perioperative triple antibiotic therapy with metronidazole, gentamicin, and either co-amoxiclav or amoxicillin. Surgery was performed by a consultant or an ST3+ equivalent grade supervised by a consultant.

All patients had macroscopic appendicitis and the majority had complicated disease; four were gangrenous or perforated and five cases had local contamination.

### Postoperative Outcomes

#### 30-Day Complication Rate

Five of seven patients had a 30-day postoperative period free of complications. Two suffered C-D II complications. The first developed a superficial wound infection after discharge requiring outpatient assessment and was treated with a course of oral antibiotics. The second required IV antibiotics postoperatively for a non-COVID-19 lower respiratory tract infection. There were no major C-D III to V complications nor COVID-19-related sequelae. No patients were readmitted.

#### Spinal Anesthesia-Related Complications

Intraoperatively, two of seven experienced pain; one aforementioned patient required IV fentanyl and the second required conversion to GA after initial skin incision due to failure of SA.

Postoperatively, no patients suffered hypotension or bradycardia requiring medical intervention. There was no incidence of puncture site infection, hematoma, urinary retention, or other adverse neurological sequelae.

#### Length of Stay

The median LOS was 1 day ( *n* = 4) with the remainder staying 2, 3, and 5 days. The patients staying 3 days or longer had perforated appendicitis and required additional IV antibiotics postoperatively.

#### Follow-Up Patient Experience Survey

All seven were followed up via telephone 30 days postoperatively. Two patients reported pain intraoperatively and the median maximal pain score within 24 hours postoperatively was seven. Five expressed overall satisfaction with the experience, however, four stated preference for GA over SA if they had the operation again. Finally, it took a median 14 days (range, 3–21) for patients to return to normal activity.

## Discussion

Open appendicectomy in adults is a relatively rare approach in the United Kingdom, with a 2017 multicenter study estimating that a laparoscopic approach was undertaken in 93% of cases. 11 Performing open appendicectomy under SA is rarer still.

We present seven patients who underwent open appendicectomy under SA which, to our knowledge, is the first such case series in an economically developed country published in the modern era. It is common and successful practice in resource-limited environments with a single-center reporting 112 cases in 1 year. 12

This was a joint strategy from the general surgery and anesthetic departments, the latter of whom regularly use SA for lower abdominal surgery in obstetrics. Our main focus was postoperative complication rates. The wound infection rate was potentially higher than the 8.6% quoted by the literature for open appendicectomy under GA. 13 This is understandable given sample size and considering that most patients had complicated disease with either gangrenous or perforated appendicitis with contamination. Despite this, no major complications were reported; it could have been predicted that our intra-abdominal collection rate would be higher than the 1.2% literature rate for open appendicectomy given the severity of cases. Remaining results depict appendicectomy under SA as feasible in terms of LOS and patient satisfaction. Conversion rate from SA to GA was also low.

Considering technical factors, we anecdotally found SA provided effective muscle relaxation with no difficulties for lavage of contamination or abdominal closure. This was reflected in favorable operative times and low postoperative collection rate. Due to the potential presence of the virus in peritoneal fluid and feces, all theater staff present wore level III personal protective equipment including filtering face piece 3 masks, water-resistant gowns, and face shields.

There are, however, limitations with the technique highlighted by this series including potential failure of neuroaxial blockade resulting in pain, as well as patient preference for GA if given choice. Consequently, wider patient acceptance of regional anesthesia for abdominal surgery may limit extensive use within the United Kingdom. Despite this, it is important to recognize that most of these cases also had a satisfactory experience and hence in extreme circumstances, we argue that SA for appendicectomy should remain an option. Although not encountered in this series, there are also specific SA complications such as significant hypotension and neurological sequelae that may further make the technique less appealing. 14

This case series is limited by small sample size but rapidly changing practices and lower than normal patient volumes prohibited larger datasets. There will be variation in how centers across the United Kingdom have interpreted the RCS guidance and altered their management of appendicitis accordingly. Nationally, this will be captured in the ongoing United Kingdom COVID-19 HAREM prospective multicentre study. 15

In line with previous studies, we agree that laparoscopic appendicectomy should remain a gold standard for appendicitis where possible. However, the direction of the COVID-19 pandemic is unknown and with the ease of lockdown measures, a second “wave” of infection remains a distinct possibility. We therefore propose that open appendicectomy under SA should be reserved as a valuable option in the arsenal of the Emergency General Surgeon. This could be of particular benefit in patients with suspected or proven COVID-19 needing appendicectomy as it provides a means to avoid the potential aerosol generation from intubation and laparoscopy. This also could remain an alternative for higher risk respiratory patients in general.
